# Long cap-assisted visualization enabling bleeding point identification and clip hemostasis for stomal variceal bleeding

**DOI:** 10.1055/a-2825-9571

**Published:** 2026-03-19

**Authors:** Nobutaka Doba, Kosuke Shibayama, Shinzo Abe, Daiki Sakuma, Masanobu Someya, Shin Maeda

**Affiliations:** 1 36998 Department of Gastroenterology, Yokosuka City Hospital, Yokosuka, Japan; 2 Department of Gastroenterology, Yokohama City University Graduate School of Medicine, Yokohama, Japan

A 72-year-old man with alcoholic liver cirrhosis had undergone transverse colostomy 4 years earlier. Over the preceding 2 years, he had been hospitalized nine times for hematochezia and hemorrhagic shock; however, bleeding ceased spontaneously after each admission, and repeated endoscopic evaluations failed to identify the source.

**Fig. 1:**
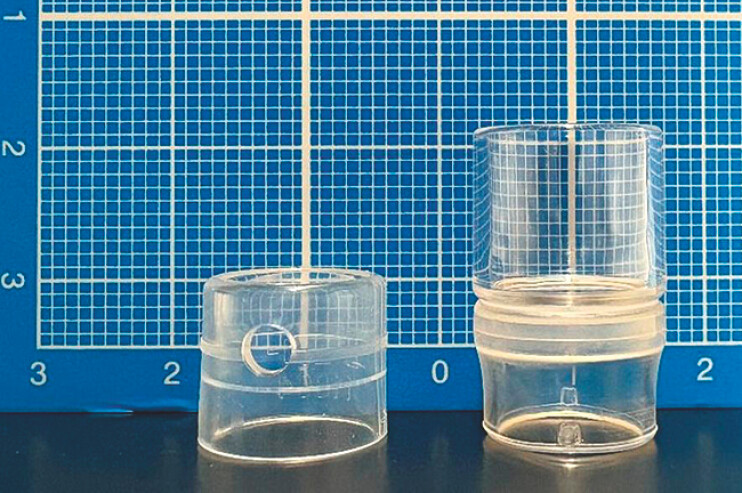
Comparison of distal attachment caps. Left: short cap with a 4-mm tip protrusion. Right: long cap with a 12-mm tip protrusion.

**Fig. 2:**
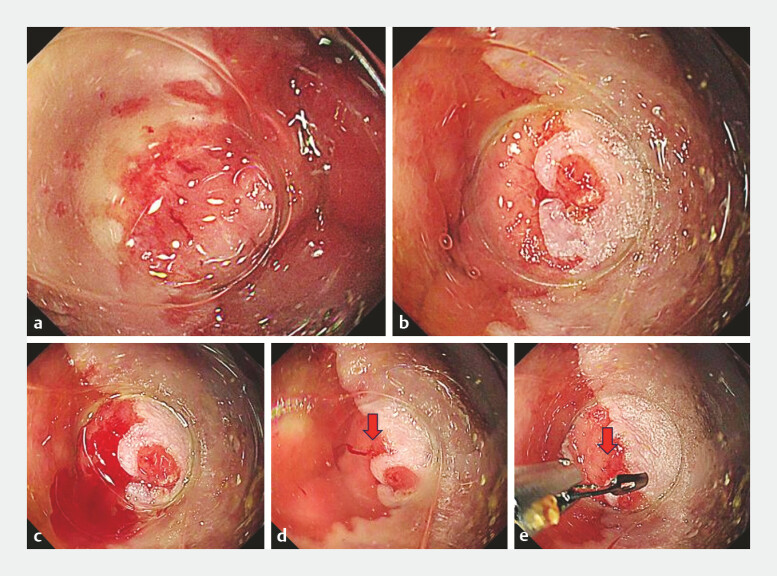
Long cap-assisted endoscopic hemostasis for stomal variceal bleeding. **a** Stomal fistula margin. Multiple dilated vessels are visible along the fistula margin. **b** Stomal fistula at the 2 o’clock position. An erosive lesion with a slight elevation is observed. **c** Bleeding from the area adjacent to the erosion. Gentle suction applied to the erosive lesion induces oozing hemorrhage. **d** Identification of the bleeding point.Water irrigation with retention of water within the cap lumen allows the clear visualization of the bleeding point. The arrow indicates the bleeding point. **e** Clip hemostasis. While maintaining the stable visualization of the bleeding point within the cap, hemostasis is achieved using a standard endoscopic clip. The arrow indicates the bleeding point.

**Fig. 3:**
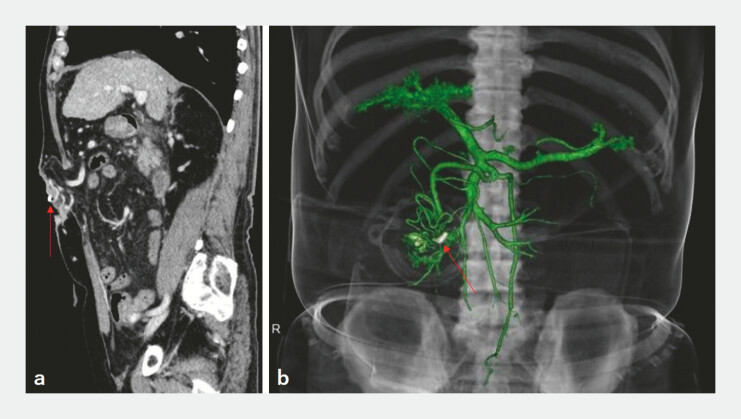
Contrast-enhanced computed tomography findings. **a** Contrast-enhanced computed tomography (portal venous phase), a sagittal view. Well-developed collateral vessels are observed immediately beneath the stomal fistula. The arrow indicates the hemostatic clip. **b** Three-dimensional vascular reconstruction. Collateral vessels originating from the superior mesenteric vein are distributed around the stomal fistula, suggesting the presence of stomal varices associated with portal hypertension. The arrow indicates the hemostatic clip.

One day after discharge, blood accumulation was noted in the stoma appliance, prompting urgent readmission. No active bleeding was observed upon inspection. Urgent colonoscopy without bowel preparation revealed normal stool without intraluminal blood, suggesting an extraintestinal source near the stomal fistula. Because the extraintestinal location hindered stable visualization, a long transparent cap was attached to facilitate endoscopic observation ( Fig. 1 ) 1 . Gentle compression stabilized the view, revealing multiple dilated vessels with a distinct erosion at the stomal margin ( Fig. 2 **a, b** ). Suction induced oozing and water immersion clearly delineated the bleeding point ( Fig. 2 **c, d** ; Video 1 ). Hemostasis was achieved with an endoscopic clip, resulting in immediate cessation of bleeding ( Fig. 2 **e** ; Video 1 ). Contrast-enhanced computed tomography demonstrated collateral vessels beneath the fistula, consistent with stomal varices ( Fig. 3 ).

Long cap-assisted clipping for stomal variceal bleeding.Video 1

No procedural complications occurred. Surgical suturing was performed, and the patient was discharged. One month later, rebleeding from a different stomal varix was successfully controlled using the same technique. At the time of writing, the patient is awaiting interventional radiological treatment for definitive portal decompression.

Stomal variceal bleeding typically requires portal decompression with a transjugular intrahepatic portosystemic shunt or embolization 2 3 . When accessible, endoscopic therapy provides rapid hemostasis as a bridge to definitive treatment. Options include injection sclerotherapy performed under direct visualization 4 and endoscopic ultrasound-guided coil or thrombin injection for deeper collaterals 5 . Mechanical clipping is effective when a discrete bleeding point is identified, particularly in extraintestinal stomal varices where direct compression is feasible.

Compared with short caps, the long cap has a longer distal extension and a wider lumen, allowing stable observation and device insertion while maintaining a clear visual field, even at the extraintestinal stomal margin ( Fig. 1 ). Although long cap-assisted hemostasis has been reported for gastrointestinal bleeding 1 , its application in stomal variceal bleeding remains rare.

Endoscopy_UCTN_Code_TTT_1AQ_2AZ
